# The Design and Rationale of a Multicentre Randomised Controlled Trial Comparing Transperineal Percutaneous Laser Ablation With Transurethral Resection of the Prostate for Treating Benign Prostatic Hyperplasia

**DOI:** 10.3389/fsurg.2021.755957

**Published:** 2021-10-18

**Authors:** Wei Zhang, Weituo Zhang, Qian Guo, Lei Chen, Zheying Meng, Yanjun Xu, Nailong Cao, Bing Hu, Biyun Qian

**Affiliations:** ^1^Department of Ultrasound in Medicine, Shanghai Jiao Tong University Affiliated 6th People's Hospital, Shanghai, China; ^2^Shanghai Institute of Ultrasound in Medicine, Shanghai Jiao Tong University Affiliated 6th People's Hospital, Shanghai, China; ^3^Clinical Research Institute, Shanghai Jiao Tong University School of Medicine, Shanghai, China; ^4^Department of Urology, Shanghai Jiao Tong University Affiliated 6th People's Hospital, Shanghai, China; ^5^Hongqiao International Institute of Medicine, Shanghai Tong Ren Hospital, Shanghai, China

**Keywords:** benign prostatic hyperplasia, urology, surgery, laser ablation, sonography

## Abstract

**Background:** Transurethral resection of the prostate (TURP) is regarded as the “gold standard” for the treatment of benign prostatic hyperplasia (BPH) in elderly men. However, ~15% of patients who had undergone TURP had intraoperative and postoperative complications, such as bleeding, urinary incontinence and urethral stricture. Transperineal percutaneous laser ablation (TPLA) is a method that places the optical fibre directly into the prostate with the guidance of ultrasound imaging, and the percutaneous transperineal approach is performed distal to the urethra and rectum to protect these structures and reduce urethral or postoperative infection. Several studies on TPLA for BPH treatment have been reported recently; however, high-quality randomised controlled trial (RCT) to evaluate its efficacy, safety, and long-term follow up remain absent.

**Methods:** This study is a multicentre, open-label RCT to assess the efficacy and safety of TPLA vs. TURP to treat BPH. We hypothesise that the TPLA has non-inferior efficacy to TURP in the International Prostate Symptom Score (IPSS) at 3 months changing from the baseline and lower incidence of post-surgery complications. One hundred and fourteen patients with BPH will be recruited at 19 sites and randomly assigned at 1:1 to TPLA or TURP groups. The patients will be followed up at 1, 3, 6, 12, and 24 months after the procedure.

**Discussion:** The study will be the first multicentre clinical trial including 16 participating centres in China, Italy, Switzerland, and Poland with relatively large sample size 114. By comprehensively compare the safety and efficacy of TPLA with TURP in patients with BPH, especially concerning the improvement of lower urinary tract symptoms (LUTS) and complication incidence, the study will help to illustrate the clinical value of TPLA and provide a beneficial alternative treatment for BPH patients.

**Clinical Trial Registration:** The study has been registered on Chinese Clinical Trial Registry (http://www.chictr.org.cn), identifier [ChiCTR1900022739].

## Introduction

Benign prostatic hyperplasia (BPH) is a common urologic disease in elderly men. Approximately 50% of men >50 years of age will have pathological evidence of BPH, with this number increasing to >80% as men reach their eighth decade of life and older (1–3). The series of clinical manifestations, such as urinary frequency, urgency and incontinence caused by BPH, are collectively referred to as lower urinary tract symptoms (LUTS) which brings many inconveniences to elderly patients' life and the decline of life quality (4–6).

Treatment options of BPH start at watchful waiting and progress through medical to surgical interventions, depending on the patients' symptoms. Currently, surgery has become the main treatment method with the progression of BPH in addition to drug therapy. Although transurethral resection of the prostate (TURP) is regarded as the “gold standard” for the minimally invasive treatment of BPH (7–9), many intraoperative and postoperative complications persist in TURP (10, 11), depending on the surgeon expertise, such as the urinary incontinence or iatrogenic injury caused by operation (12–14). The tissue on the cut surface forms an eschar, whose organisational structure is fuzzy, leading to injury even perforation of the surgical capsule. In addition, the eschar may bring about secondary haemorrhage (15) after shedding and repeated haematuria after the operation. And the high temperature of TURP can cause urethral burn and postoperative urethral stricture (16–21).

With the emergence of various intracavitary minimally invasive devices, new technologies are being applied to the medical field, such as transurethral enucleation of the prostate (TUEP), which including plasma kinetic enucleation of the prostate (PKEP), holmium laser enucleation of prostate (HoLEP), thulium laser enucleation of the prostate (ThuleP) and some other laser based methods (22). Lasers show better haemostatic effects and cause less bleeding during operations (23–26). Additionally, lasers are non-conductive; thus, they can be applied in patients with heart pacemakers and other electric implants. Although these new techniques might offer sufficient efficacy and lower risk of complications over standard bipolar-TURP (27, 28), However, the levels of evidence are too low and follow-up still too short to offer solid recommendations, and larger comparative studies are needed to evaluate the ultimate impact of the en-bloc approach on postoperative outcomes (22, 29). And since the surgical approach are transurethral avoiding injury is challenging, and haematuria and urethral stricture may occur after the operation (30–33).

With the guidance of high-frequency transrectal ultrasound (TRUS), which is widely used in the diagnosis and treatment of BPH, the clinician can accurately locate the position of the lesion in the prostate and guide the puncture through the image in real time. The percutaneous transperineal approach occurs distal to the urethra and rectum, to protect these structures and reduce the difficulty of preoperative preparation and operation, and lower postoperative infection effectively (34).

Can laser treatment of prostate guided by TRUS achieve the minimally invasive treatment of BPH using a percutaneous transperineal approach? Researchers have verified transperineal percutaneous laser ablation (TPLA) a feasible and safe method (35–40). Pacella CM conducted a single arm trial, performed the analysis on 160 patients with 6 months follow and 83 patients with 12 months after TPLA (41), and de Rienzo G conducted TPLA for 21 patients, verified the significant advantage in Qmax, IPSS, and peculiar ability to preserve ejaculation (38). However, high-quality RCTs, compared with TURP, the most widely used surgical method and “gold standard” for BPH patients, remained absent. Additionally, the efficacy and safety should be illustrated by a longer follow-up time.

This study aims to evaluate the following: the efficacy and safety of TPLA to treat BPH compared with TURP; to explore the clinical value including the prognosis and curative effect of TPLA after long-term follow-up; to seek scientific advice to optimise the relevant parameters and precautions of TPLA treatment and; to provide evidence for the indication of TPLA by analysing the advantages and disadvantages in subgroups.

## Methods and Analysis

### Study Design

This study is a multicentre, open-label, randomised controlled clinical trial. This study protocol follows the standard protocol for clinical trials in accordance with the SPIRIT 2013 statement and follows the CONSORT statement for clinical trial transparency (42, 43). The study is registered on Chinese Clinical Trial Registry (http://www.chictr.org.cn), registration number: ChiCTR1900022739. The study was approved by the Independent Ethics Committee of Shanghai Jiao Tong University Affiliated Sixth People's Hospital (Ethics number: 2018-034) and the local ethics committee of participating centres.

### Objective and Hypothesis

The primary objective of the study is to assess the efficacy and safety of TPLA vs. TURP in treating BPH. We hypothesise that TPLA has non-inferior efficacy to TURP according to the IPSS score change at 3 months from baseline and lower incidence of post-surgery complications. Secondary endpoints, including the maximum urinary flow rate, patients' quality of Life (QoL) and post-urination residue, operation time and hospitalisation time, will also be assessed.

The secondary objective is to determine the optimal target population in patients with BPH for TPLA treatment.

### Participants

#### Patient Recruitment

**Figure 1:**
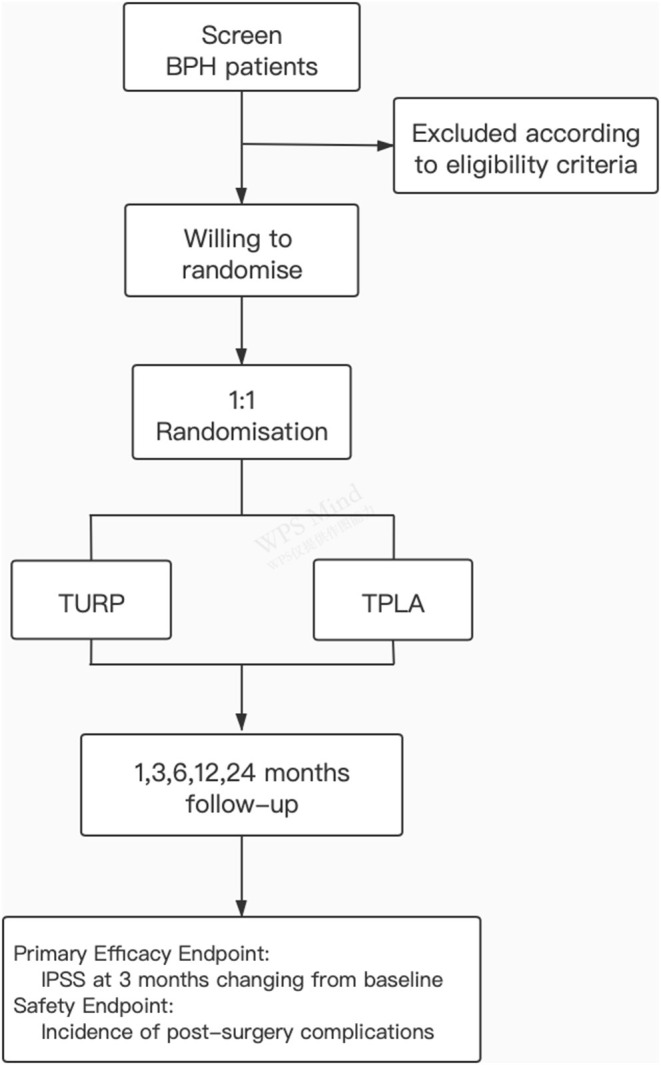
Subject diagram. The flow diagram of this randomised controlled trial.

The participants will be recruited from patients with BPH at Shanghai Sixth People's Hospital and other participating centre hospitals. Patients with LUTS and imaging-confirmed BPH and who are willing to accept surgical treatment could be recommended by clinicians, sonographers or advertisement, and instant messaging software (WeChat). The subject diagram is presented in Figure 1. Before the recruitment the research staff would introduce the two operation methods (TPLA and TURP) including the strengths and drawbacks, respectively, to the participants, and clarify the risks and potential benefits of participating the trial. The screening and randomisation procedure, the operation, hospitalisation, and the follow-up details would be fully discussed as well. And written informed consent will be obtained before eligibility screening. The participants can decide to withdraw their consent for any reason during the entire study. The results of follow-up will be disseminated to the study participants through paper reports and telephone communication.

#### Patient Screening

Eligibility screening will be performed in 14 days before surgery, as listed in Table 1.

**Table 1:** Contents of enrolments, interventions, and assessments.

**Stage**	**Screening**	**Preoperative examination**	**Surgery**	**Hospitalisation**	**Follow-up**				
**Time**	**−14**~−**1 days**	**−7**~−1** days**	**0 day**	**4 ± 3 days**	**1 month**	**3 months**	**6 months**	**12 months**	**24 months**
Informed consent	√								
Medical history	√								
Vital signs		√	√	√					
ECG		√	√	√^*^					
Blood routine	√		√	√^*^					
Urine routine	√		√	√^*^	√	√	√	√	√
Coagulation function	√		√	√^*^					
Liver and kidney function		√	√	√^*^					
Blood electrolyte		√	√	√^*^					
Visual analogue score (VAS) pain score				√(24 h after surgery)					
PSA	√				√	√	√	√	√
Qmax	√				√	√	√	√	√
Urodynamics		√				√			
IPSS	√				√	√	√	√	√
QoL score	√				√	√	√	√	√
EQ-5D score	√				√	√	√	√	√
Transrectal ultrasound	√				√	√	√	√	√
MRI	√					√			
Transabdominal bladder ultrasonography	√				√	√	√	√	√
Sexual function (IIEF-5)	√				√	√	√	√	√
Ejaculatory dysfunction (MSHQ-EjD)									
Operation time			√						
Specific time of resection or ablation			√						
Intraoperative blood loss and blood transfusion records			√						
AE records			√	√	√	√	√	√	√
Catheter retention time				√					
Hospitalisation				√					

##### Inclusion Criteria

1. Patients with LUTS caused by BPH who have failed prior treatment or are unsuitable for medical treatment as judged by the clinician.
2. Male older than 50 years.
3. IPSS score ≥8.
4. Prostate volume measured by TRUS between 30 and 100 ml.
5. A maximum urinary flow rate (Qmax) ≤15 mL/s.
6. Transabdominal bladder ultrasonography: post-urination residue ≥50 ml.
7. Provide Informed consent.

##### Exclusion Criteria

1. Urethral stenosis.
2. Previous prostate, bladder, or urethral surgery.
3. Dysuria caused by bladder dysfunction.
4. History of a long-term indwelling catheter.
5. BPH with mainly medial lobe hyperplasia (intravesical prostate projection ≥10 mm); transabdominal bladder ultrasonography revealed bladder calculi or obvious bladder tumour.
6. Patients with pathology-proven prostate cancer; Serum prostate specific antigen (PSA) >4 ng/ml and PSAD ≥0.15 ng/ml^2^, no iatrogenic injury.
7. Patients with known neurological disorders—e.g., multiple sclerosis, Parkinson's disease, or known history of spinal cord injury.
8. Post-rectal surgery or patients with anal atresia.
9. Patients with severe coagulation disorders or with infection.
10. Patients participating in another clinical study or who have been enrolled in another clinical study 4 weeks before randomisation.

##### Withdrawal Criteria

The participants can quit between the signing of informed consent and random assignment because of any of the following reasons:

1. Participants do not meet with the eligibility criteria.
2. Participants decide to withdraw the informed consent.
3. Researchers consider participants unsuitable to continue with the study.

### Procedures

#### Treatment Allocation and Blindness

Confirmation of the study eligibility is required before patients are randomly assigned to receive treatment. The investigator will review the screening procedure and confirm that the patient meets all the inclusion criteria and does not meet any of the exclusion criteria. Subjects willing to randomise will be enrolled in the RCT cohort and assigned to the TURP or TPLA treatment at 1:1 randomly. Computer-generated random numbers applied. The stratified block randomisation will be applied using the Interactive Web Response System (IWRS; URL: https://jcri.shsmu.edetek.cn/actims_ngm_V2/login.action), which was designed by Clinical Research Institute of Shanghai Jiao Tong University School of Medicine (Shanghai, China). The stratification factors include study sites and severity of BPH (according to the baseline IPSS score, 8–19 as moderate, and 20–35 as severe). Study sites will conduct competitive enrolment with four as the minimum sample size requirement. Because the trial is open-label, both the patients and the surgeon are aware of the treatment allocation, but the outcome evaluations are performed by blinded readers.

#### Intervention

After enrolment, urologists, sonographers, and radiologists will perform TURP and TPLA according to the treatment allocation.

##### TPLA

**Figure 2:**
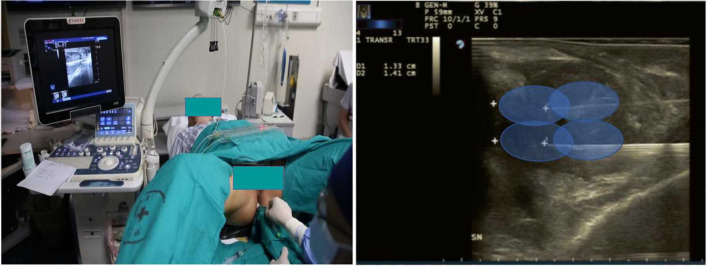
Patient position and needle positioning. The needle was inserted into prostate under guidance of TRUS and performed oval thermal ablation scope.

**Figure 3:**
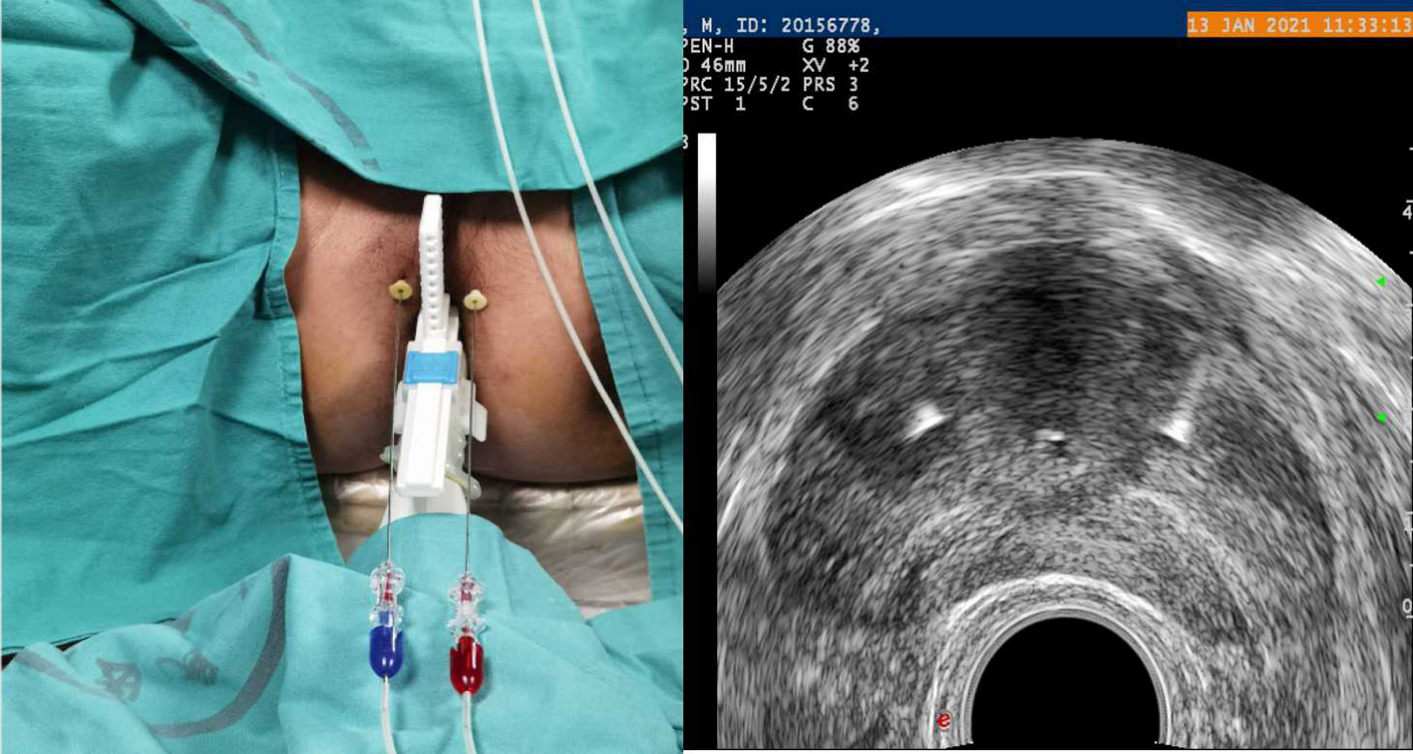
Planning of the procedure. Two fibres for a prostate volume smaller than 40 ml (one for each side).

**Figure 4:**
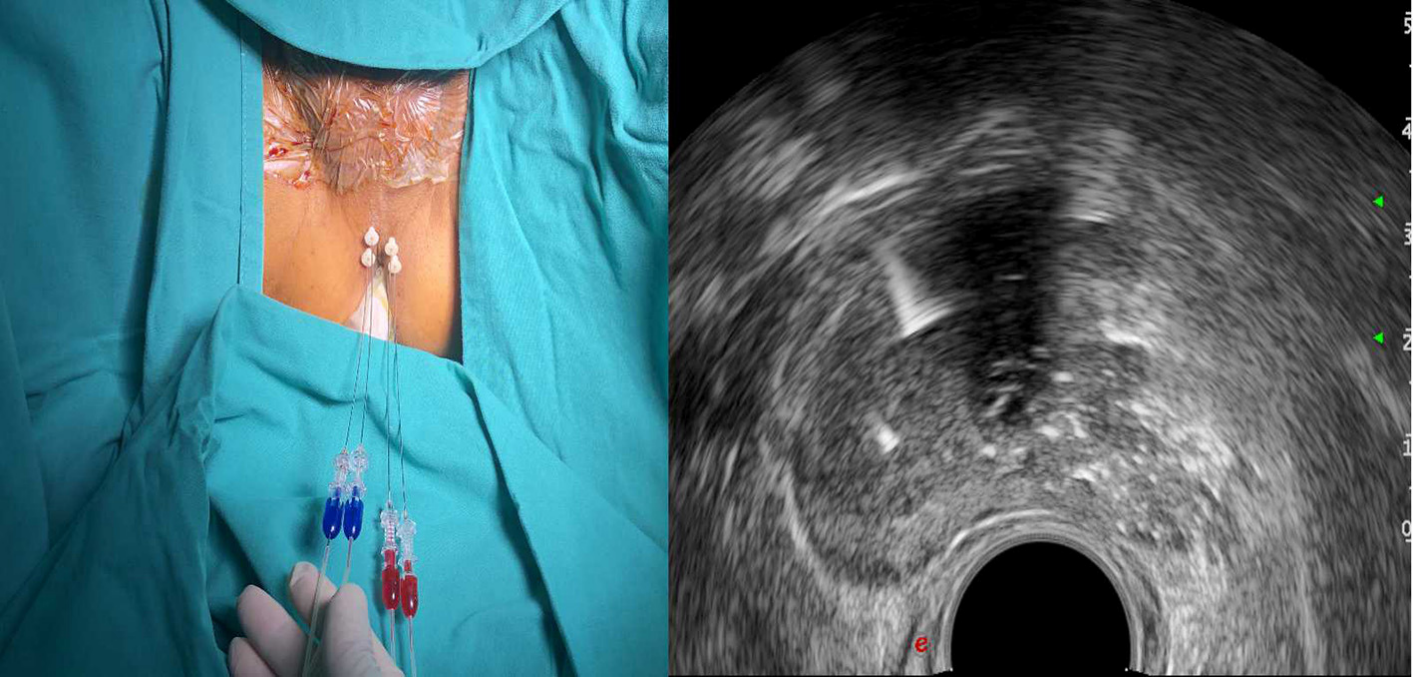
Planning of the procedure. Four fibres for a prostate volume larger than 40 ml (two for each side).

The patient will be placed in the lithotomy position on a treatment couch with a three-cavity urethral catheter inserted and will undergo routine disinfection and draping before surgery. The treatment will be performed under local anaesthesia in the perineal region with lidocaine. The optical fibre will be inserted inside the prostate under TRUS guidance (Figure 2). For a prostate volume smaller than 40 ml, two fibres will be used, while four fibres will be used for a prostate volume larger than 40 ml (Figures 3, 4). A safe distance of 8 mm from the fibre to the urethra will be maintained, at least 15 mm from the fibre tip to the bladder neck and 10 mm from the outer edge of the prostate capsule. In the case of two applicators, they must be positioned sequentially at a mutual distance of 8–10 mm. Each treatment will be performed at a fixed power of 3 W. Each ablation time is 600 s to maintain the total energy equal to 1,800 J per fibre. The main goal of the treatment is to deliver 1,800 J of energy for the first ablation; if needed, the pull-backs can deliver a total amount of 1,200–1,800 J, depending on the prostate size. At the end of the treatment, 8 mg of dexamethasone can be administered intravenously for anti-oedema and anti-inflammatory reactions.

##### TURP

The patient will be placed in the lithotomy position. After anaesthesia and regular disinfection, 26-F continuous-flow resectoscope with a standard tungsten wire loop (Olympus) will be inserted into the prostate. Images of the bladder and ureterostma will be recorded, and withdraw the resectoscope to the verumontanum to inspect the prostate. The two lateral lobes and the medial lobe of the prostate hyperplasia can be displayed. An Olympus UES-40 SurgMaster (Olympus Europa Holding GmbH) electrical current generator was used with settings of 280 W for cutting and 120 W for coagulation. Both the medial lobe and two lateral lobes of the prostate will be resected. The procedure should be carried out to the bladder neck and not surpassing the verumontanum, after sufficient haemostasis and urethral inspection. The images will be recorded and a three-cavity urethral catheter will be inserted for continuous bladder irrigation.

##### Hospitalisation and Observation

After the treatment, the patients should be closely supervised for 2 days with standard post-operation care and the catheter, in the absence of adverse events, is removed within 2 weeks after the procedure.

### Follow-Up

The efficacy and safety endpoints will be collected 1, 3, 6, 12, and 24 months after the operation, as listed in Table 1.

### Measurements and Clinical Outcomes

#### Data Collection

The measurements and data collection schedule are presented in Table 1.

The general medical history of each patient will be obtained through interviews and a review of existing medical records. The data collected and recorded include demographics (e.g., age, gender, and race/ethnicity) and meaningful past or current illnesses. A history of known drug allergies, other allergies, or a history of allergic reactions and all concomitant medications (including Chinese medicine) will also be recorded.

Vital signs (heart rate, respiration, and blood pressure), electrocardiograph (ECG), amount of bleeding and laboratory tests (liver and kidney function, routine blood tests, and blood electrolytes) will be evaluated before and after the operation within 24 h, and the operation time, treatment time, intraoperative blood loss, and blood transfusion will be recorded as well. The post-operation observation include urine routine, urine flow rate test, IPSS, transabdominal bladder ultrasonography (residual urine measurement), and TRUS, quality of life QoL score, European five-dimensional health scale (EQ-5D) score, sexual function evaluation International Index of Erectile Function-5 (IIEF-5), Male Sexual Health Questionnaire-Ejaculatory Dysfunction (MSHQ-EjD) score. Enhanced magnetic resonance imaging (MRI) will be performed 3 months after treatment.

Regarding quitting patients, the researchers will collect the medical materials integrally and record their quitting reasons and time.

All of the data collectors and evaluators will be blinded to the group assignments. The data in this trial will be collected using electronic case report forms (eCRFs) using a remote data capture (RDC) system (https://clinicaldata.shsmu.edu.cn/). The RDC system will be provided by the Clinical Research Institute of Shanghai Jiao Tong University School of Medicine (Shanghai, China).

#### Adverse Events, Serious Adverse Events, and Safety Measurements

Any clinically significant post-treatment changes determined by the investigator or a designee, including vital signs, ECG, and the safety endpoint within 3 months after surgery should be recorded as AEs.

SAEs include more serious urethral stenosis or urinary incontinence after the operation that results in longer hospitalisation, physical disability or even death.

Any AE or SAE will be monitored and then reported. The severity and causality should also be analysed. The data submitted will be monitored by the Ethics Committee of Shanghai Jiao Tong University Affiliated Sixth People's Hospital and the local ethics committee. Any SAE should be immediately reported to the aforementioned ethics committee.

#### Clinical Outcomes

##### Efficacy Endpoint

The primary efficacy endpoint is the IPSS score change at 3 months from baseline.

The secondary efficacy endpoints include:

1. IPSS change at 6, 12, and 24 months from baseline.
2. Qmax at 3, 6, 12, and 24 months after treatment.
3. Residual urine volume at 3, 6, 12, and 24 months after treatment.
4. Quality of life score, IIEF-5 score, MSHQ-EjD score (3 item and bother) at 3, 6, 12, and 24 months after treatment.
5. European five-dimensional health scale score EQ-5D at 3 and 6 months after treatment.

##### Safety Endpoint

The safety endpoint is the incidence of complications within 3 months after surgery (hyponatremia, urinary retention, TURS, erectile dysfunction, bladder neck contracture, urethral stricture, perforation of the prostate capsule, infection, sepsis, urinary incontinence, bladder rupture, haematuria, and persistent bacteriuria for more than 3 weeks after surgery), intraoperative blood loss; VAS pain score; postoperative urinary catheter retention time; surgical success rate.

##### Health Economics Assessment

The comparison of operation time and hospitalisation time are included.

### Statistical Considerations

#### Sample Size Calculation

The sample size is calculated regarding the non-inferiority of efficacy. We assume the mean IPSS changes at 3 months from baseline in the TPLA and TURP groups are the same, the standard deviation of both groups is σ = 6, and the non-inferiority margin is Δ = 3 (10, 34, and 37). With α = 0.05 and β = 0.2, a sample size of 102 is required. Considering a drop-off rate of 10%, the overall sample size is 114.

#### Statistical Methods

We will report our results according to the CONSORT 2010 Statement (44).

The demographic and clinical characteristics of subjects in TPLA/TURP arms will be described. The qualitative variables will be presented as absolute numbers and proportions. Quantitative variables will be presented as the means ± SD or medians and IQRs as appropriate.

The primary endpoint, the IPSS score change at 3 months from baseline, will be compared using the Mann–Whitney *U*-test or Student's *t*-test as appropriate. The safety endpoint, the incidence of complications within 3 months after surgery, will be compared using the χ^2^ test. The primary analysis will be performed on the ITT set defined as all patients who meet the eligibility criteria and undergo randomisation. Predefined subgroup analysis includes the age group, BPH severity according to the baseline IPSS score, the sites grouped by country. Missing values will be imputed with multiple imputations under the assumption of missing at random except for the safety endpoints. All statistical analyses will be performed using R (version 3.5.1).

## Discussion

This study is a multicentre randomised clinical trial to assess the efficacy and safety of the novel surgery technique TPLA, which may provide an alternative treatment with lower surgery complications for properly selected BPH patients. With a relatively large sample size, this study intends to achieve internal and external validity at the same time. TRUS-guided TPLA provides a new method for patients with BPH with moderate or severe LUTS. Using very thin applicators and high-precision energy delivery, percutaneous laser ablation has been demonstrated to be safe and effective (45–48). Several preliminary studies including short-term follow-up proved its feasibility, Pacella revealed IPSS improved from 22.5 ± 5.1 to 7.7 ± 3.3 at 6 months and 7.0 ± 2.9 at 12 months, Cai HJ proved ultrasound guided TPLA effective and safe with the QoL improved from 4.9 ± 1.7 to 2.3 ± 1.3 and the Qmax improved from 8.5 ± 3.0 to 15.2 ± 4.8 mL/s at 6 months after the procedure (37, 41). However, no comparison with patients treated with other techniques has been performed, especially TURP, the most widely used surgical method and “gold standard” for BPH patients. This clinical trial will focus on comparing TPLA with TURP, evaluating the results with efficacy, safety index, and health economics assessment.

The use of TRUS guidance with a biplane probe allows repositioning of applicators during the manoeuvre based on the physician experience, and the amount of energy is planned in advance according to the baseline volume of the prostatic lobes (35, 38, 49). The assessment of the extent of the coagulation zone after ablation with transrectal contrast-enhanced ultrasonography is considered a very useful tool for early result assessment (50–52). Therefore, ultrasonography plays an important role in the study. With the monitor of real-time TRUS picture, the laser fibre could be placed precisely in the location and be adjusted dynamically. TRUS guidance contributes to reduce the possibility of urethral injury, and contrast-enhanced ultrasonography after TPLA is helpful to evaluate the dimension and evolution of coagulation, and a supplementary ablation could be conducted if necessary (53).

To the best of our knowledge, the sample size is much larger than most of the previous studies. The randomisation, standard follow-up procedure, and blinding of raters are adopted to increase internal validity. The multicentre, international cooperation are adopted to increase external validity.

TPLA provides a new method of operation, but the indication is relatively narrow compared with TURP. Patients with bladder dysfuntion (38), rectal surgery history, an oversized prostate with a prominent medial lobe or BPH with cystolith would not be appropriate for TPLA. As a matter of fact, it's an supplementary option for BPH patients, instead of a replacement to other surgery method. The procedures should be performed by very experienced interventional physicians with sufficient knowledge of US-guided thermal ablation, which could be a limitation for the technique to be generalised. Technically speaking, the skill and technique levels in each centre vary, so our experienced experts and professors would provide instruction to ensure successful TPLA proceeds before official recruitment, and a teaching video would be transmitted to investigators. Each centre is requested to successfully accomplish at least two TPLA operations independently before the official enrollment. During the procedure, the images and operation videos would be recorded and transmitted to the supervisors for quality control. Another limitation is that the IPSS and complication confirmation may be subjective; thus, our independent blind evaluators would be indispensable.

This randomised controlled multicentre clinical trial comprehensively compare the safety and efficacy of TPLA with TURP in patients with BPH, especially concerning the improvement of LUTS, the patients' QoL and complication incidence. The results of this study will help to illustrate the clinical value of TPLA and provide a beneficial alternative treatment for BPH patients.

## Ethics Statement

The studies involving human participants were reviewed and approved by Independent Ethics Committee of Shanghai Jiao Tong University Affiliated Sixth People's Hospital. The patients/participants provided their written informed consent to participate in this study. Written informed consent was obtained from the individual(s) for the publication of any potentially identifiable images or data included in this article.

## Author Contributions

WeiZ, WeitZ, and ZM designed the study and drafted the manuscript. QG, LC, YX, and WeitZ revised the study protocol. NC participated in the recruitment and patients screening in urology department. BH and BQ approved the final version of the manuscript, provided administrative support, have full access to all the data in the study, and have final responsibility for the decision to submit for publication. All authors have read and approved the final manuscript.

## Funding

BH received Shanghai Key Discipline of Medical Imaging Fund (No. 2017ZZ02005) and Shanghai Key Discipline of Clinical Imaging Fund (No. shslczdzk03203), and financed the study, including advertising and general publicity expense, the laboratorial analyses and consumables of participants.

## Conflict of Interest

The authors declare that the research was conducted in the absence of any commercial or financial relationships that could be construed as a potential conflict of interest.

## Publisher's Note

All claims expressed in this article are solely those of the authors and do not necessarily represent those of their affiliated organizations, or those of the publisher, the editors and the reviewers. Any product that may be evaluated in this article, or claim that may be made by its manufacturer, is not guaranteed or endorsed by the publisher.

## Abbreviations

BPH: benign prostatic hyperplasia
TPLA: transperineal percutaneous laser ablation
TURP: transurethral resection of the prostate
RCT: randomised controlled trial
RDC: remote data capture
LUTS: lower urinary tract symptoms
IPSS: International Prostate Symptom Score
IWRS: Interactive Web Response System
QoL: quality of life
EQ-5D: European five-dimensional health scale
VAS: visual analogue score
ECG: electrocardiograph
PSA: prostate specific antigen
TRUS: transrectal ultrasonography
MRI: magnetic resonance imaging
AE: adverse event
SAE: serious adverse event
IIEF-5: International Index of Erectile Function-5
MSHQ-EjD: Male Sexual Health Questionnaire-Ejaculatory Dysfunction
eCRF: electronic case report form.
